# Polystyrene Microplastic Interferes with Yolk Reserve Utilisation in Early *Artemia salina* Nauplii

**DOI:** 10.3390/toxics13080700

**Published:** 2025-08-20

**Authors:** Chiara Maria Motta, Chiara Fogliano, Marco Trifuoggi, Maria Toscanesi, Anja Raggio, Simona Di Marino, Paola Venditti, Gianluca Fasciolo, Bice Avallone, Rosa Carotenuto

**Affiliations:** 1 Department of Biology, University of Naples Federico II, 80126 Naples, Italy; mottacm@unina.it (C.M.M.); simona.dimarino@unina.it (S.D.M.); paola.venditti@unina.it (P.V.); gianluca.fasciolo@unina.it (G.F.); bice.avallone@unina.it (B.A.); rosa.carotenuto@unina.it (R.C.); 2 Department of Chemical Sciences, University of Naples Federico II, 80126 Naples, Italy; marco.trifuoggi@unina.it (M.T.); maria.toscanesi@unina.it (M.T.); 3 Stazione Zoologica Anton Dohrn, 80122 Naples, Italy; anja.raggio@szn.it; 4 NBFC, National Biodiversity Future Centre, 61, 90133 Palermo, Italy

**Keywords:** carbohydrate characterization, hatching rate, naupliar growth, oxidative stress, volatile organic compounds (VOCs)

## Abstract

Polystyrene microfragments are among the most common plastic pollutants globally. They significantly affect aquatic life, harming various organs and tissues. In this study, we examined the effects of 3 µm polystyrene beads (MPs, 20 µg/L) on development and yolk resorption in pre-feeding nauplii of *Artemia salina*, a lecithotrophic crustacean used in toxicity testing. Results showed a reduced hatching rate, slower growth, and the onset of oxidative stress. Histological analysis revealed no significant morphological alteration; however, yolk platelets lost N-acetyl galactosamine (galNAc), and resorption was delayed. Lectin staining also showed a reduction in N-acetyl glucosamine (glcNAc) in the gut brush border, indicating impaired gut function. Gas chromatography detected the release of nanogram amounts of toxic volatile compounds (VOCs, ethylbenzene, xylene, benzaldehyde, and styrene) into the culture medium. In conclusion, the data demonstrate a delay in larval yolk resorption that can likely be attributed to the release of VOCs, which induce oxidative stress. Further research is urgently needed, given the potential biological and ecological implications of this finding.

## 1. Introduction

The increasing production of plastic over the past few decades has created a significant global issue, as millions of metric tons of waste are discarded into the aquatic environment each year [1]. Once in the water, plastic waste eventually breaks down into microfragments of various shapes and sizes (microplastics, <5 mm, and nanoplastics, <500 nm), which disperse in the water column and accumulate in sediments [1,2].

Several studies have addressed the issue of microplastic sampling methods [3,4], reported concentrations [5], and highlighted the effects caused by fragments of various types, sizes, shapes, and concentrations [6]. Microplastics are ingested by vertebrates [7,8] and invertebrates [9,10], particularly zooplanktonic species and larvae [11,12].

Once internalised, microplastics cause oxidative stress [13] and the lipid peroxidation damages membrane functionality [14]. Interference is also registered on the DNA [15] and gene expression [16] with consequences at the cellular to organismic levels. Processes affected include immune response [17], metabolism [18], reproduction [19], and behaviour [20].

Microplastics are transported to tissues through the bloodstream [21]. It has been demonstrated that they can also reach and accumulate in the maternal yolk, and from there, be transferred to developing embryos with effects that are not yet fully understood [22]. There is also limited information about the direct effects of MPs on yolk resorption in embryos and the implications for development. 

This study, therefore, aimed to examine the effects of 3 µm microbeads in polystyrene (MPs; 20 µg/L; [23,24]) on yolk reserve consumption in embryos and pre-feeding nauplii of *Artemia salina*. This lecithotrophic crustacean is widely used as a model organism for toxicity studies [24,25] since data obtained can be applied to other zooplanktonic larvae. As primary consumers, they feed on microalgae and are susceptible to ingesting microplastics, which are mistaken for prey, thereby facilitating the transfer of contamination to higher trophic levels in the food chain [26].

## 2. Materials and Methods

### 2.1. Experimental Design

The first step was to determine whether MP exposure affects development, in particular, hatching rate and early naupliar growth [27,28]. The interference with naupliar anatomy and yolk distribution was examined in nauplii in toto and histological sections [29,30]. Particular focus was given to the presence and distribution of N-acetylglucosamine and N-acetyl galactosamine, carbohydrates essential for larval development, as they are significant components of the lipovitellin complex of yolk granules [31]. 

In an initial attempt to clarify the mode of action, it was considered that polystyrene releases styrene and other pro-oxidant volatile organic compounds (VOCs) [32,33,34]. Their presence in naupliar culture media was therefore measured using gas chromatography, and the potential oxidative effects on embryos and nauplii were evaluated by measuring lipid peroxidation, susceptibility to oxidative stress, ROS levels, and total antioxidant capacity. 

The effects were examined on encysted embryos and early pre-feeding nauplii. For embryos, treatments were carried out on intact, chorionated, and dechorionated (decapsulated) cysts. On nauplii, experiments were conducted to confirm that the observed effects were independent of feeding on beads. The presence of MPs in the gut was also examined, both in whole larvae and in sections. These tests were necessary because the literature reports contradictory effects of bead ingestion, ranging from no effects [35] to a severe impact on growth due to energy depletion over time [36].

### 2.2. MPs and Reagent Characterisation and Analytical Methods

#### 2.2.1. Polystyrene Bead Characterisation

Polystyrene beads (MPs), 3 µm diameter (standard deviation < 0.1 µm, coefficient of variation < 3%), obtained from Sigma Aldrich (Milan, Italy), were examined for roundness and integrity using a scanning electron microscope. Briefly, samples were placed on an Isopore membrane filter with a pore size of 0.4 µm and mounted on ultra-pure aluminium pin stubs. After platinum sputter coating with a Leica EM ACE200, samples were analysed with a Jeol field-emission scanning electron microscope, JSM 6700-F (Akishima, Tokyo, Japan). Digital images were analysed using ImageJ version 1.54k, following the method described by de Oliveira et al. [37], to verify integrity, size, and shape homogeneity.

Microbead aggregation in seawater cultures and attachment to the external cyst wall were excluded by fluorescence microscopy after staining with the lipophilic dye Nile Red (1 mg dye in 1 mL of acetone [38,39]). In brief, 1 mL of dye solution was added to 5 mL of culture suspension; after 5 min in the dark, small aliquots were collected, placed on a watchmaker’s slide, and covered with a coverslip [40]. Fluorescence was observed at 40× magnification using a Leica Axioskop microscope (Wetzlar, German) equipped with a UV lamp.

#### 2.2.2. Gas Chromatography Analysis of Naupliar Culture Media

Volatile organic compounds (VOCs) released from polystyrene microbeads were determined in seawater used for naupliar culture using a gas chromatograph GC-MS 5977B MSD (Agilent Technologies, Santa Clara, California, USA) equipped with a DB-624 Ultra Inert capillary column (30 m × 250 µm × 1.4 µm). The gas chromatograph was operated in split mode, and the separation was conducted with an oven temperature programmed as follows: an initial setting of 35 °C (3 min hold), followed by a ramp to 200 °C at 10 °C/min. The injector was held at 180 °C. The mass spectrometer was operated in SIM mode. The GC–MS was interfaced with an automated purge and trap unit (PT) (ATOMX Teledyne Tekmar, Mason, Ohio, USA) directly connected to the GC column through a heated transfer line. The 2–3 mL of samples were closed in vials using silicone-Teflon septa and loaded on the autosampler. In the vial, purging was performed by a He flow rate of 40 mL/min for 20 min. During purging, sample vials can be heated to 40 °C.

The limit of detection (LOD) and quantification (LOQ) were determined by analysing 10 replicates of a matrix fortified at a low concentration near LOD. The limits of quantification are included in the range of 0.001 to 0.005 µg/L. The recovery percentage was verified using certified matrices (QCM-115, QCM-116, Ultrascientific, Bologna, Italy), and the calculated average values ranged from 80% to 110%.

#### 2.2.3. Oxidative Damage in Encysted Embryos and Newborn Nauplii

Lipid hydroperoxides (HPs) were measured in homogenates obtained from encysted embryos (18 h post-hydration) and newborn nauplii (18 h post-hatching). Aliquots containing 10 μg of protein were diluted in a 0.1 M monobasic phosphate buffer, pH of 7.4 [41]. The decrease in NADPH absorbance, resulting from the combination of reactions facilitated by glutathione peroxidase and glutathione reductase enzymes, was monitored at a wavelength of 340 nm in the presence of GSH. The results were expressed as nmol NADPH oxidised per minute per milligram of protein.

The susceptibility to oxidative stress was assessed in vitro by determining variations in hydroperoxide levels [42]. Iron and ascorbate (Fe/As) were added to 10% homogenate at 100/1000 μM concentrations for 10 min at room temperature. The reaction was halted by adding 0.2% of 2,6-di-t-butyl-p-cresol (BHT). The hydroperoxides were measured using the same methods described above.

The reactive oxygen species (ROS) were determined by measuring the conversion of 2′,7′-dichlorodihydrofluorescin diacetate (DCFH-DA) to the fluorescent compound dichlorofluorescein (DCF) [43]. Homogenate aliquots (25 μg of proteins) were mixed with 200 μL of a 0.1 M monobasic phosphate buffer at a pH of 7.4, containing 10 μM DCFH-DA. FeCl3 was added to a final concentration of 100 μM, and the mixture was incubated for 30 min. The conversion of DCFH-DA to the fluorescent product DCF was measured using a microplate reader (Tecan Infinite 200 pro plate reader) with excitation and emission wavelengths of 485 nm and 530 nm, respectively. The conversion of DCFH to DCF in the absence of homogenate was also measured to determine the background signal. A standard curve of DCF was utilised to calculate the amount of DCF formed in picomoles.

The overall capacity of cells to counteract oxidative stress (ABTS) was assessed by incubating the non-radical form of ABTS overnight with potassium persulfate (245 mM). The decolourisation of the formed ABTS+ radical (2,2′-azinobis-(3-ethylbenzothiazoline-6-sulfonic acid)) caused by cellular antioxidants was then measured at a wavelength of 734 nm. A calibration curve was created using a solution of 3,5-Di-tert-4-butylhydroxytoluene (BHT). The total antioxidant capacity was expressed as the equivalent amount of BHT per milligram of protein.

### 2.3. Test Organism Handling and Endpoints Measured

#### 2.3.1. Care and Treatment of *Artemia salina*

*Artemia salina* cysts were obtained from commercial sources and selected based on their high hatching rate (>90%). Dried cysts were placed in glass tanks filled with artificial seawater (Instant Ocean Sea salt, Blacksburg, Virginia, USA; salinity 36‰; [44]) or artificial seawater containing polystyrene microbeads. Static cultures (without water changes during exposure) were maintained at 22 ± 1 °C under a natural photoperiod with gentle agitation. Nauplii were fed twice daily with yeast and a commercial preparation containing unicellular organisms and algae. The experiments were discarded if the mortality rate in control samples exceeded 10% at 2 days [44]. Whenever necessary, the nauplii were collected using a wide-mouthed plastic pipette to prevent damage, and a light source was employed [45]. Microplastic suspensions were prepared by diluting 20 µL of the stock solution (1 mg/mL in water) in 1 L of seawater (1.35 × 10^6^ beads/L; 20 µg; [46]).

#### 2.3.2. Cysts Decapsulation

Decapsulation (dechorionation) was conducted as described by Sorgeloos et al. [47]. Briefly, 50 mg of cysts were hydrated in 25 mL of mineral water for 1 h and treated with 25 mL of sodium hypochlorite for 10 min. The decapsulated cysts were then rinsed repeatedly in mineral water, transferred to seawater, and treated as described in Section 2.3.1. Decapsulated cysts retained a high hatching rate (around 75%); neonate nauplii were viable and showed no signs of morphological or functional distress.

#### 2.3.3. Hatching Test

An average of 106 ± 16 cysts was placed into sterile wells containing 3.5 mL of pure seawater or seawater with polystyrene beads (20 µg/L). Each sample was prepared in quadruplicate, and the experiments were repeated in triplicate using different batches of cysts. Hatching occurred 30–36 h after hydration began; sampling was conducted 12 h after the first swimming nauplius emerged from the control culture.

At sampling, the cultures were recovered and filtered through an *Artemia* sieve. Cysts and nauplii were resuspended in Falcon tubes with 5 mL of 75% ethanol to halt development and examined under a Leica Axioskop stereomicroscope at 5× or 10× magnification to count the total number of cysts and nauplii (at the L0/L2 stage). The ratio of nauplii to cysts, expressed as a percentage, was calculated by multiplying the ratio by 100. The staging was carried out according to Copf et al. [48] and previous observations made in our laboratory [49].

#### 2.3.4. Naupliar Growth

A total of 250 mg of cysts was placed in 500 mL of seawater and allowed to develop as described in Section 2.3.1. To evaluate growth, nauplii were collected daily for 3 days after hatching. Aliquots containing approximately 1000 to 1500 individuals were transferred into Falcon tubes and fixed with 4% formalin. The nauplii were rinsed in 75% ethanol to remove formalin residues and examined under a light microscope for staging. The number of nauplii at different stages divided by the total number of nauplii examined, multiplied by 100, gave the percentage composition of the population. An increased or decreased frequency of nauplii at late stages indicated an acceleration or delay in developmental progression. Growth was also assessed; larval length was measured from digital photographs (n = 75 randomly selected nauplii per treatment) using the software ImageJ 1.8.0. Growth was expressed as the percentage increase in length during the transitions from stages L0 to L1, L1 to L2, and L2 to L3. All are non-feeding stages relying on maternal reserves.

#### 2.3.5. Polystyrene Beads Feeding

Control newborn nauplii were fasted for 12 h, then fed fluorescent beads (Fluoro-Max Green Fluorescent Particles; Thermo Fisher, Fremont, California, USA) for 8 h, and examined in toto under UV light. Alternatively, nauplii were maintained as described in Section 2.3.1 and then processed for light microscopy (see Section 2.3.6). Sections were stained with Nile Red to reveal ingested MPs. The staining solution (Sigma-Aldrich, Milan, Italy) was prepared as a stock solution by dissolving 25 mg of Nile Red in 100 mL of 0.4% acetone. A working concentration of 5.0 µg/L was achieved by further diluting with water [50]. Sections were stained for 15 min in the dark, washed, and observed under UV light.

#### 2.3.6. Histological Investigations

Nauplii were collected from cultures using a plastic Pasteur pipette, fixed in Bouin’s solution (15 mL picric acid, 5 mL formaldehyde, and 1 mL acetic acid) for 5 min, and then dehydrated through a graded series of ethanol (75%, 95%, and absolute; a total of 20 min). For embedding, they were cleared in xylol for 5 min and then transferred to paraffin at 60 °C for 30 min, with two changes. Serial sections (6 µm) were stained with hemalum–eosin to show general morphology or with fluorescein-conjugated lectins to localise carbohydrates. In particular, WGA (*Triticum vulgaris* agglutinin) lectin detected N-acetyl glucosamine (glcNAc), while SBA (soybean agglutinin) lectin detected beta-linked N-acetyl galactosamine (galNAc) [51]. These sugars are carbohydrate components of the yolk globules [31]. A total of 1 µL of lectin (Vector Laboratories Inc, Burlingame, California, USA; 2 mg/mL) was diluted in 19 µL of PBS buffer, pH of 7.2–7.4. The solution was immediately applied to the deparaffinised and rehydrated sections and left in the dark for 15 min in a humid chamber at room temperature. After rinsing in PBS buffer, the sections were examined with an Axioskop light microscope equipped with a UV lamp. Labelling was defined as positive or negative by the same observer. Negative controls were prepared by incubating sections with the lectins and the specific competing sugar, or by omitting the lectin in the reaction to check for autofluorescence. Naupliar staging was carried out as described above (Section 2.3.3).

The percentage of polystyrene-treated nauplii with persistent yolk granules was determined at stages L4, L5, and L6. One hundred fifty nauplii per stage and treatment were examined in different sections. They were considered positive if they showed more than ten granules per 100 µm^2^ of tissue.

### 2.4. Analysis of Data

Numerical data for hatching rate, growth progression, and naupliar length were expressed as the mean value of treatment samples/the mean value of the control samples * 100 [52]. All values obtained were analysed for significance by one-way ANOVA or t-Student tests using GraphPad-Prism 8 software (GraphPad Software, Inc., San Diego, CA, USA). Cohen’s d was used to determine the size effects of treatments. Values are reported in text and legends if effects are statistically significant (*p* < 0.05).

## 3. Results

### 3.1. Polystyrene Beads Characterisation

**Figure 1:**
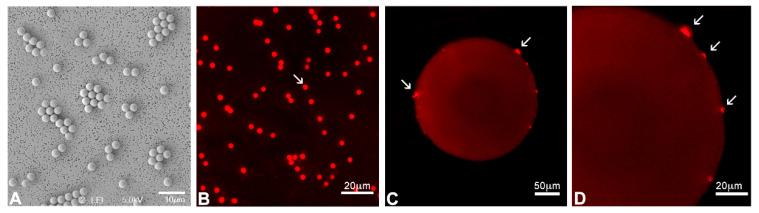
Characterisation of polystyrene beads (3 µm diameter). (**A**,**B**) Beads’ size and shape are regular. (**C**,**D**) Cysts show occasional fragments of unidentified material (arrows) adhering to the wall. In toto, at the scanning electron microscope (**A**) or after in toto staining with Nile Red and UV light illumination (**B**–**D**).

Scanning electron microscopy revealed that the polystyrene microbeads (3 µm nominal diameter) were uniformly spherical, with smooth surfaces and no evidence of fractures, deformation, or surface irregularities (Figure 1A,B). Measured diameters were consistent with the manufacturer’s specifications (standard deviation < 0.1 µm, coefficient of variation < 3%). Nile Red fluorescence staining confirmed the hydrophobic nature of the beads and showed no detectable aggregation in seawater cultures. Moreover, no attachment of the microbeads to the external cyst wall was observed under fluorescence microscopy (Figure 1C,D).

### 3.2. Effects of MPs on Hatching and Growth

**Figure 2:**
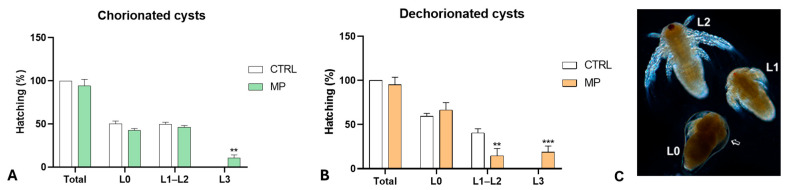
Hatching in chorionated (**A**) and dechorionated (**B**) *Artemia salina* cysts exposed to polystyrene microbeads (48 h of hydration). Hatching percentage (total) and per cent composition of the naupliar population (control, CTRL; treated, MPs). In the treated populations, note the presence of stage L3 nauplii, which are absent in the controls. (**, *p* < 0.01; ***, *p* < 0.001). (**C**) Nauplii at L0 to L2 stages. The newborn nauplius is still enveloped by a membrane (arrow). In toto, unstained nauplii observed under incident light. Magnification: 10×.

After exposure to MPs, the hatching percentage did not change compared to controls in both intact (Figure 2A) and dechorionated cysts (Figure 2B). In controls, the population was always composed of L0 to L2 nauplii (Figure 2C). In contrast, after MP exposure, a significant percentage of L3 nauplii was present. The effect size for treated samples was >10. The development of hatched nauplii was observed three days after hatching.

**Figure 3:**
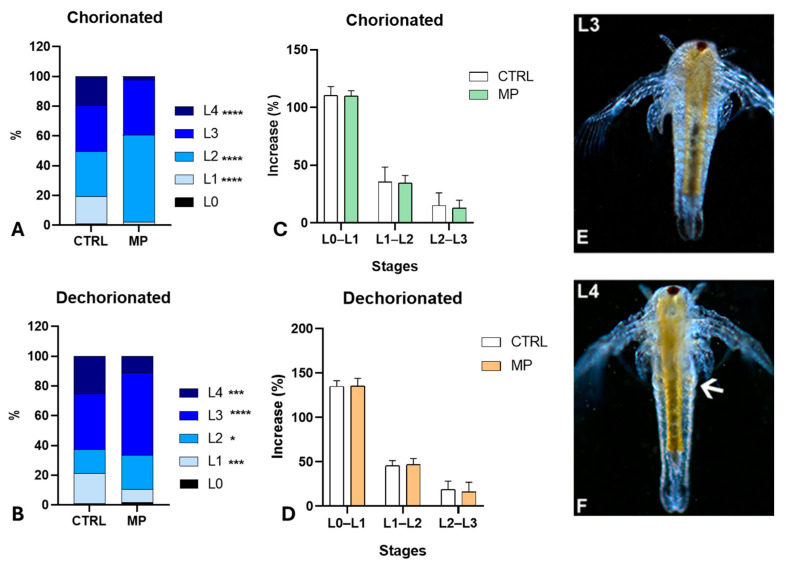
Growth of *Artemia salina* nauplii obtained from intact (**upper panel**) or dechorionated (**lower panel**) cysts (3 days post-hatching). (**A**,**B**) Composition of the naupliar population. The significant decrease in the percentage of L4 nauplii indicates that MPs induced a delay. (*, *p* < 0.05; ***, *p* < 0.001; ****, *p* < 0.0001). (**C**,**D**) Length increase (per cent) in nauplii progressing from stage L0 to L1, L1 to L2, or L2 to L3. (**E**,**F**) Nauplii at the L3 and L4 stages; thoracopod buds (arrows). In toto, unstained nauplii observed under incident light. Magnification 5×.

In control populations from intact cysts (Figure 3A), approximately 18% and 19% of nauplii were at the L1 or L4 stages, while about 30% and 31% of the nauplii were at the L2 and L3 stages, respectively. Following exposure to MPs, there was a significant decrease in nauplii at the L4 stage (approximately 2%) and an increase in nauplii at the L2 stage (about 58%), indicating a delay in development. The nauplii at the L3 stage (roughly 37%) showed no significant change compared to the controls. In all samples, the effect size exceeded 5.3.

In samples obtained from dechorionated cysts (Figure 3B), polystyrene exposure also induced a significant delay, as demonstrated by the increased percentage of L3 stage nauplii and the decreased percentage of L4 stage nauplii compared to controls. Consequently, variations were found in the percentages of L2 and L1 stage nauplii (effect size > 2.8). After MP exposure, no significant differences were noticed in the naupliar growth rate. Progression from stage L0 to L1, L1 to L2, and L2 to L3 determined a body length increase that did not differ in control and MP-treated larvae, regardless of whether obtained from chorionated (Figure 3C) or dechorionated (Figure 3D) cysts.

### 3.3. Effects of Polystyrene on Naupliar Anatomy

**Figure 4:**
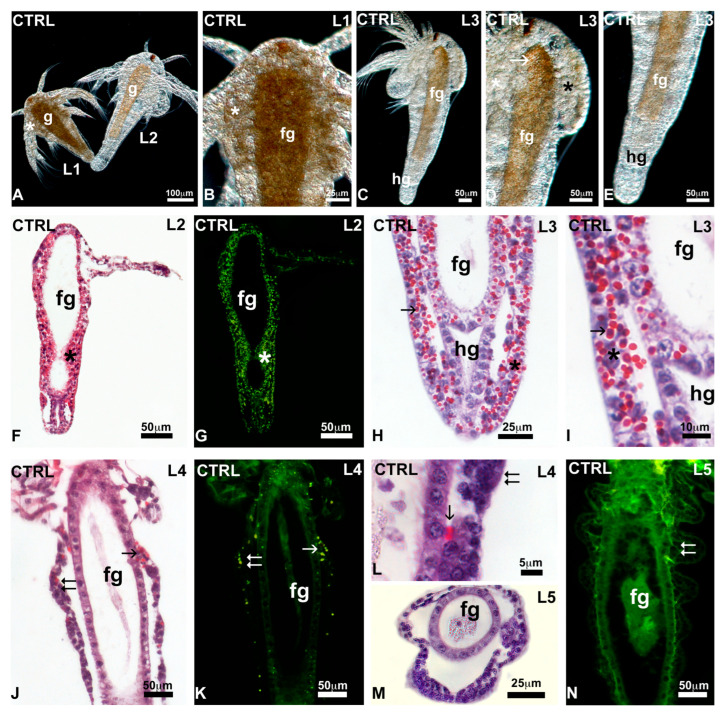
Distribution of reserves in *Artemia salina* nauplii. (**A**,**B**) Presence of reserve in the gut (g) and body wall (*). (**C**–**E**) Reduced presence in the foregut (fg) and body wall (*); absence in the hindgut (hg). Transparent fat globules (small arrows). In toto, unstained nauplii observed under incident light. (**F**,**G**) Diffuse presence of eosinophilic, autofluorescent yolk globules (*). Foregut (fg), body wall (*). (**H**,**I**) Details showing the eosinophilic globules (arrows) in the foregut (fg) epithelium and body wall (*). Note the hindgut (hg) epithelium with no yolk. (**J**,**K**) Nauplius with thoracopod buds (double arrows) and a few eosinophilic, autofluorescent yolk globules (arrows). (**L**) Detail of a thoracopod bud (double arrow) with a residual yolk globule (arrow). (**M**,**N**) Transverse and frontal sections of nauplii with no yolk globules. Thoracopod (double arrow). Foregut (fg). Staining with hemalum–eosin; observation in bright field (**F**,**H**–**J**,**L**,**M**) or under UV light (**G**,**K**,**N**) to highlight the autofluorescence of eosin.

Control nauplii observed in toto, at stages L0 (Figure 2C), L1 (Figure 2C and Figure 4A,B), and L2 (Figure 2C and Figure 4A), showed a dense body, rich in fats. By stage L3 (Figure 3E and Figure 4C), the body was transparent, and lipids were present only in the foregut (Figure 4D). The hindgut (Figure 4E) was completely transparent. 

In histological sections, control nauplii at the L2–L3 stages (Figure 4F–I) showed distinct fore and hindgut, lined by a monolayered epithelium. The body wall and gut epithelium contained numerous yolk globules, intensely stained in red by eosin (Figure 4F,H,I); when observed under UV light, they appeared intensely autofluorescent in green (Figure 4G). In nauplii at the L4 stage, characterised by thoracopod buds (Figure 4J), the number of yolk globules was drastically reduced (Figure 4J–L). Yolk globules were completely absent in nauplii at the stage L5 (Figure 4M,N).

**Figure 5:**
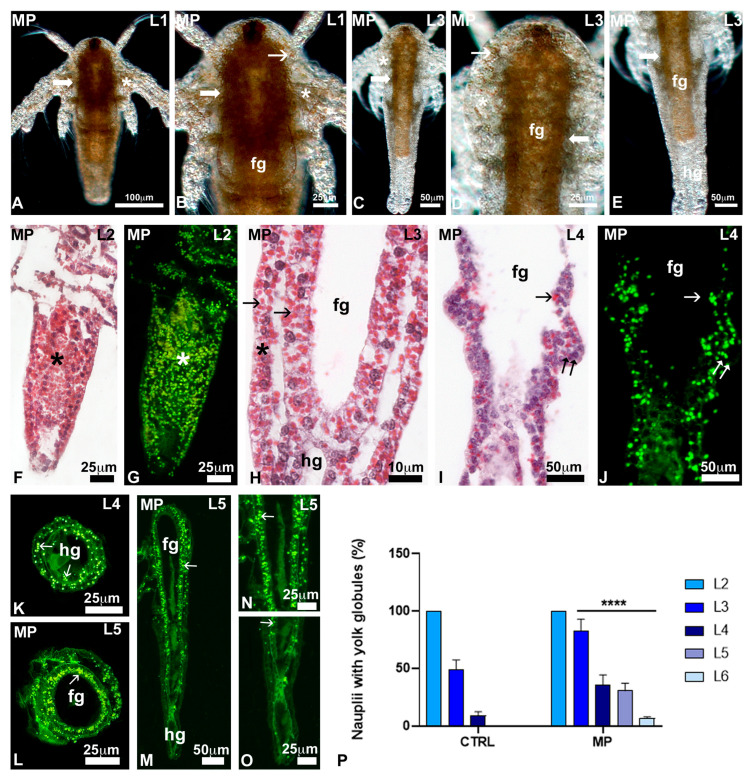
Distribution of reserves in early-stage nauplii exposed to 3 µm polystyrene beads. (**A**–**E**) Presence in foregut (fg) and body wall (*) but not in the hindgut (hg). Notice the dense gut wall (large arrows). Fat globules (small arrows). In toto, unstained nauplii observed under incident light. (**F**,**G**) Diffuse presence of eosinophilic, autofluorescent yolk globules (*). (**H**) Detail of the body wall showing the eosinophilic globules (arrows) in the foregut (fg) epithelium and body wall (*). (**I**,**J**) Nauplius with thoracopod buds (double arrow) and many eosinophilic, autofluorescent yolk globules (small arrows). (**K**) Hindgut (hg) with many dispersed, residual yolk globules (arrows). (**L**) Many yolk globules (small arrow) concentrate in the gut wall. (**M**–**O**) Yolk globules (small arrow) concentrate in the foregut wall. (**P**) Percentage of L2 to L6 stage nauplii with yolk globules; (****, *p* < 0.0001). Staining with hemalum–eosin; observation in bright field (**F**,**H**,**I**) or under UV light (**G**,**J**–**O**) to highlight the autofluorescence of eosin.

After exposure to MPs, no significant changes were noticed in L1 nauplii (Figure 5A): they were rich in lipid reserve, at the level of the alimentary canal and the body wall (Figure 5B). In the nauplii at the L2 and L3 stages, the reserve was still present in the body wall (Figure 5C,D) and the foregut, concentrated in the wall (Figure 5C–E). In histological sections, all nauplii at the L2 (Figure 5F,G) and 82.5% of nauplii at the L3 stage (Figure 5H,P) contained many yolk globules dispersed throughout the body. Unlike controls, the presence of globules remained significant in 35.4% of nauplii at the L4 stage (Figure 5I–K) and in 30.4% and 5.5% of nauplii at the stage L5 (Figure 5L–P) and L6, respectively. In these latter, globules tended to concentrate in the wall of the foregut (Figure 5L,N).

### 3.4. Effect of MPs on Carbohydrates—Staining with FITC-Conjugated SBA and WGA Lectins

**Figure 6:**
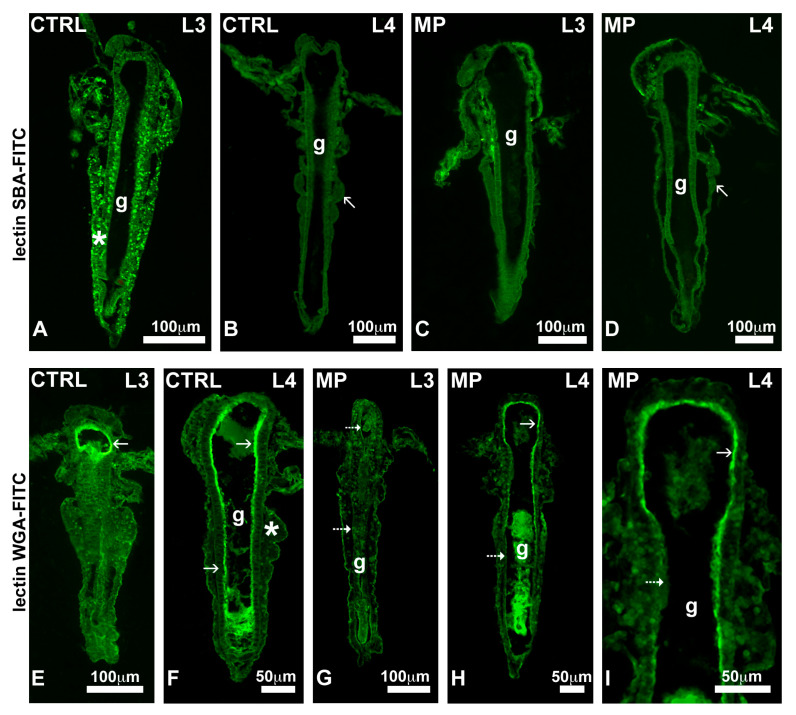
Distribution of N-Acetyl galactosamine (upper panel) and N-Acetyl glucosamine (lower panel) in nauplii exposed to 3 µm polystyrene beads. (**A**) Labelled yolk globules in the body wall (*). (**B**–**D**) Unlabelled nauplii. Thoracopod buds (arrows). (**E**,**F**) Gut wall with intensely labelled brush border (arrows). (**G**) Completely unlabelled brush border (dotted arrow). (**H**) Nauplius with labelled (arrow) and unlabelled (dotted arrow) brush border. (**I**) Detail. Gut (g). Sections were stained with FITC-conjugated SBA or WGA lectins.

In controls, the lectin SBA, specific for galNAc, intensely stained yolk globules present in L1 to L3 stage nauplii (Figure 6A). As expected, in stage L4 nauplii, devoid of yolk, no significant staining was detected (Figure 6B). In nauplii exposed to MPs, the lectin never stained the yolk, no matter the stage examined (Figure 6C,D).

In controls, the lectin WGA, specific for glcNAc, intensely stained the brush border of the intestinal wall in all nauplii, no matter the stage (Figure 6E,F). After exposure to MPs, the brush border was unstained in L3 nauplii (Figure 6G) and was discontinuously stained in nauplii from the L4 stage (Figure 6H,I).

### 3.5. Effects of Polystyrene Feeding on Growth

**Figure 7:**
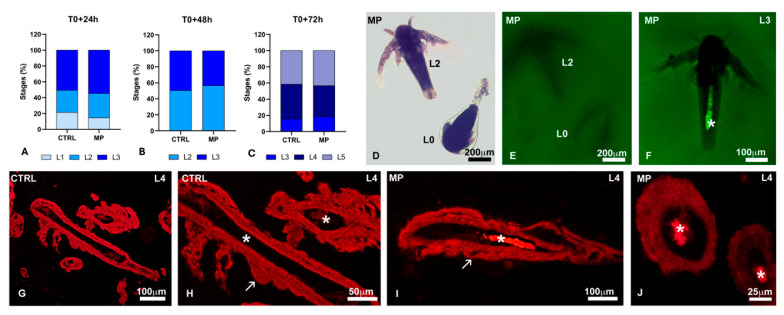
Effects of polystyrene bead ingestion on naupliar growth. (**A**–**C**) No significant effects are observed on growth in nauplii exposed at hatching (T0) for 24, 48 or 72 h. (**D**,**E**) No polystyrene beads are present in the gut of L0–L2 nauplii (pre-feeding stages). (**F**) Polystyrene beads (*) in the gut of an L3-stage nauplius. Absence (**G**,**H**) and presence (**I**,**J**) of polystyrene beads (*) in the alimentary canal of stage L4 nauplii. Thoracopod buds (arrows). (**D**) In toto, bright field observation; (**E**,**F**) in toto, observation under UV light; (**G**–**J**) histological sections stained with Nile Red and observed under UV light.

In populations treated at hatching (T0) for 24 (Figure 7A), 48 (Figure 7B), or 72 h (Figure 7C), there were no significant variations in the percentages of nauplii at the different stages compared to the respective controls. In nauplii in toto (Figure 7D–F) and in histological sections (Figure 7G–J), polystyrene beads were detected in the alimentary canal only starting from the L3 stage.

### 3.6. Effect of MPs on Oxidative Stress

**Figure 8:**
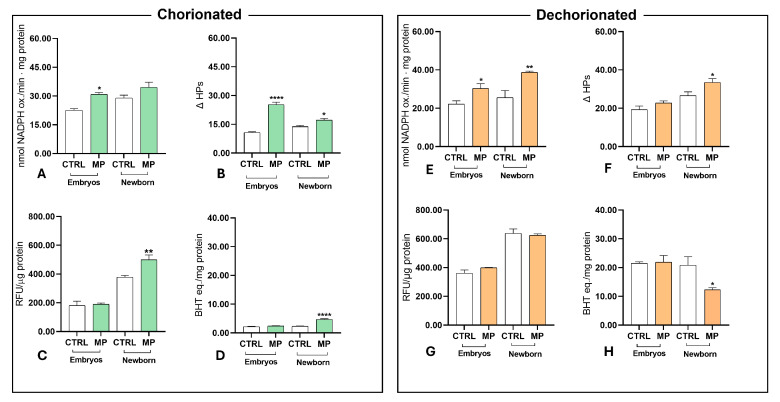
Oxidative stress in encysted embryos and newborn nauplii obtained from chorionated (**left panel**) or dechorionated (**right panel**) cysts. Hydroperoxides (**A**,**E**), susceptibility to stress (**B**,**F**), ROS content (**C**,**G**), and total antioxidant capacity (**D**,**H**). Data are averages of six determinations of different homogenates obtained from pooled cysts or nauplii populations. (*, *p* < 0.05; **, *p* < 0.01; ****, *p* < 0.0001).

In embryos obtained from chorionated cysts (left panel), the hydroperoxides (Figure 8A) and susceptibility to stress (Figure 8B) increased significantly after MP exposure. In contrast, ROS content (Figure 8C) and total antioxidant capacity (Figure 8D) showed no significant changes. In newborn nauplii, susceptibility to stress (Figure 8B), ROS content (Figure 8C), and total antioxidant capacity (Figure 8D) all increased significantly following exposure to microplastics.

In embryos from dechorionated cysts (right panel), only hydroperoxides (Figure 8E) increased significantly after microplastic treatment. In newborn nauplii, hydroperoxides (Figure 8E) and susceptibility to stress (Figure 8F) increased, while total antioxidant capacity (Figure 8H) decreased significantly due to microplastics.

### 3.7. VOCs in Culture Medium

No volatile organic compounds were detected in control seawater samples; in contrast, microgram concentrations of ethylbenzene, xylenes, and styrene were detected in the seawater where MPs were added. Traces of methyl styrene and benzaldehyde were also found (Table 1).

## 4. Discussion

The first evidence collected is that MPs did not affect the hatching percentage but accelerated embryo development. The newborn nauplii reached the L3 stage, whereas the control nauplii were still at the L2 stage. Currently, it is impossible to determine when and how the interference was exerted. The dormant *Artemia* embryo, in fact, after rehydration, progresses through three phases (differentiation, emergence, and hatching; [53]), each characterised by specific morphological and metabolic traits. It is unclear why mortality did not increase consequently, as expected [54].

Investigations also revealed that MPs induced significant oxidative stress in embryos. The result is unexpected since the shell is a continuous structure permeable only to water and gases [55], which is unlikely to have allowed beads of 3 µm diameter to contact the embryos. The obvious conclusion is that the MPs acted at a distance, via the release of diffusible chemical(s) [56]. Notably, embryos developed in dechorionated cysts exhibited a higher total antioxidant capacity, indicating a more robust protective response, likely due to the facilitated passage of these chemicals across the thinner wall [47].

The possibility that oxidative stress was a consequence of beads adhering to the cysts was excluded based on direct observations at the microscope and the fact that contrary to *Danio* chorion, in the *Artemia* cyst wall, there are no pores whose patency may be obstructed, causing hypoxia [57,58].

The chemicals released would be the VOCs [59,60,61,62], which were detected in naupliar seawater using gas chromatography. More information about the kinetics of such release is needed to accurately evaluate the effects these chemicals have on the embryo and subsequent larval development [63,64]. Toxicity, however, may have been relevant since VOCs are pro-oxidant, resulting in genotoxic and embryotoxic effects. In particular, ethyl benzene and benzaldehyde induce apoptosis [61], inflammation [65], and retard growth [66]. At the same time, xylene and benzene alter lipids, proteins, and nucleic acids [67,68].

Styrene was also detected in the culture seawater, another unexpected result, as polystyrene monomers are reported to be released from weathered plastics exposed to the disruptive effects of UV light and high temperatures [34]. The concentration (1.5 µg/L) was low compared to the 20 µg/L that can be found in nature [69]; however, styrene is potentially mutagenic [70], and its effects should not be underestimated, especially if associated with those exerted by the other VOCs.

Among the possible causes of the observed effects, leachates were not taken into consideration. Evidence indicates that they are released by aged MPs, over extended hydration periods and under conditions of high temperatures and vigorous agitation [57]. In our experiments, pristine MPs were used for a brief duration at temperatures below 22 °C, with mild agitation.

As reported, for example, in larval sea urchins [71], crustaceans [72], and fish [73], MPs induced oxidative stress also in *Artemia* embryos and nauplii. In the former case, no significant variations were observed in ROS and total antioxidant capacities. A low efficiency in defence mechanisms at this stage aligns well with the low and discontinuous metabolism associated with *Artemia* embryo development. It remains to be explained why a reduced hatching rate does not accompany an increase in hydroperoxides and susceptibility to stress but, instead, by an increased developmental rate (the increased presence of L3 nauplii).

ROS increased in nauplii, along with increased total antioxidant capacities; hydroperoxides and susceptibility to stress remained at normal levels, indicating that the detoxifying and antioxidant systems were efficient. This explains why mortality did not increase, but it remains unclear why a delay in growth was observed together with alterations in the brush border. It should be remembered that the data refer to the embryos/nauplii in toto. Different tissues/organs may have responded to the oxidative insult with different intensities. As it is impossible to examine a single individual, immunocytochemical investigations are ongoing on sections to localise and quantify the different stress markers at the cell level.

Significantly, the delay was not accompanied by variations in size. Although nauplii from different batches were slightly different in size compared to their respective controls, they exhibited comparable growth rates as they progressed from one stage to the next. These results, along with the absence of significant mortality, indicate that the nauplii were healthy but had stalled their developmental program, data that is in line with the genotoxic effects of VOCs.

To discriminate the effects of MPs on embryos and nauplii, a parallel experiment was conducted in which exposure started after hatching. The results indicated that development proceeded normally, at least until stage L4. The conclusion is that the developmental delay observed in animals exposed from hydration was caused by interferences in embryogenesis. The mechanisms of action may be multiple, especially if cytotoxic and genotoxic VOCs are the main responsible source [67,74,75,76,77]. Xylene and ethylbenzene might have altered gene expression by modifying DNA methylation [78,79]. In *Artemia salina*, acute and chronic exposure to polystyrene results in the upregulation or downregulation of 721 genes [25].

The contribution of MP ingestion to delayed development was also evaluated. Beads have potential mechanical effects [74] and/or, once in the gut lumen, may release VOCs, causing severe local toxicity [80]. Histological investigations demonstrated that the number of beads ingested is modest, not sufficient to induce fasting or worse, occlude the lumen. In contrast, staining with the lectin WGA indicated that the gut epithelium had an abnormal brush border, which was deficient in N-acetylglucosamine. Gut alterations are common in larval models exposed to toxicants [42,52,81]. In the case of polystyrene microplastics, impaired microvilli formation, epithelial cell exfoliation [18], and dysbiosis [74] have been reported [82]. Based on the available data, it was impossible to establish whether the brush border was absent/reduced or simply devoid of glcNAc and, therefore, invisible since it remained unstained. Ongoing investigations at the transmission electron microscope will clarify this point. However, alterations at the level of the brush border are indicative of energy problems [82,83] and thus justify the observed delayed naupliar development.

An interesting piece of evidence emerging from the analysis of nauplii in toto and section is that a delay in the consumption of larval reserves accompanies delayed growth. *Artemia* embryos are rich in yolk and lipid globules [84], which make the body wall of early nauplii very dense and yellowish in appearance. With progressive consumption, by the end of stage L3, the reserves are depleted, and the nauplii begin feeding. By stage L4, their body wall is entirely diaphanous. In animals exposed to MPs, this did not occur until stage L6.

Light microscopy procedures preserved yolk proteins, highlighted by eosin staining, but extracted most lipids. Their fate in development, therefore, remains to be determined. The hypothesis is that resorption was probably anticipated to support the energy requirements of larval growth. A preliminary analysis by Raman spectroscopy attempted to clarify whether this was the case, but it was unsuccessful due to the abundance of naupliar carotenoids. Transmission electron microscopy studies are therefore ongoing.

There are no reports in the literature on the interference of MPs on yolk resorption; however, decreased yolk-conversion efficiency has been observed, for example, after cadmium [85] and geraniol [86] exposures and after co-exposure to polystyrene nanoplastics and PCB-153 [87]. The causes of poor yolk resorption can be multiple. The most obvious in *Artemia* nauplii is that by releasing genotoxic VOCs, the MPs interfered with the translation/transcription of enzymes responsible for yolk degradation, cathepsin-like proteases, for example, a family of evolutionarily conserved and highly specialised proteins [88]. In zebrafish larvae, yolk retention has been linked to the up- or downregulation of many genes classified into metabolism and digestive system categories [86]. These include key genes regulating proteolysis, carbohydrate, and lipid metabolism. The observed decrease in N-acetyl galactosamine supports this possibility. Further studies with a panel of lectins will highlight changes in other typical yolk carbohydrates, such as mannose and fucose [89]. Ongoing biochemical, molecular, and electron microscopy investigations will provide further insights into whether MMP interferes with yolk degradation by altering lysosomes and the condition of the gut epithelium.

## 5. Conclusions

In conclusion, the data show that polystyrene microbeads disrupt yolk resorption in pre-feeding nauplii, causing developmental delays. This effect is associated with the loss of glcNAc in yolk globules, damage to the gut epithelium’s brush border, and oxidative stress. The release of VOCs in the culture seawater was also detected, and a correlation with the observed results is suggested.

Further studies are essential to verify these findings, to fully understand the kinetics of such release, and to identify the interference on various developmental stages. Microplastics have contaminated all aquatic environments, and their capacity to disrupt the consumption of yolk reserves poses a serious threat to the reproductive potential of many lecithotrophic species.

## Figures and Tables

**Table 1:** Total concentrations (ng/L) of volatile compounds (VOCs) released by 3 µm polystyrene beads in the seawater used for naupliar culture.

VOC	Seawater with Beads (ng/L)
Benzene	<1
Toluene	<1
Ethylbenzene	400 ± 10
Xylenes	300 ± 80
Styrene	1.500 ± 400
Methyl styrene	8 ± 2
Ethyl styrene	<1
Benzaldehyde	10 ± 3

## Data Availability

Data are available from the corresponding author upon reasonable request.
